# The Hospital World

**Published:** 1910-11-19

**Authors:** 


### The Hospital World.

### A HOSPITAL SECRETARY AS MAYOR.

The Mayoral Election for Coronation Year is attended with considerable interest to hospitals and hospital officials; but the appointment of a hospital secretary to fill the Mayoral chair is of peculiar interest. The election of Mr. W. T. Patrick, F.C.I.S., J.P., as Mayor of Guildford, is the cause of great satisfaction in the borough, and hospital officials generally will congratulate their colleague on his attainment of the highest municipal honour which his native town can confer.

Councillor Patrick's name was the only one submitted to the Council, and his appointment to the Mayoral chair was unanimous.

The eldest son of Mr. James Cranstone Patrick, of Guildford, Mr. W. T. Patrick was born in 1868. He was in 1883 appointed Assistant Secretary to the West Surrey General Benefit Society, and later became General Secretary. He has long been associated with the philanthropic life of the borough, being Hon. Secretary of Committees in the Diamond Jubilee year of Queen Victoria, and the Coronation of King Edward VII.; he has for thirteen years acted as Hon. Secretary of the Saturday Hospital Committee. He has been responsible for the organisation of the Children's New Year Parties, which have been so highly successful in the borough; while he has played a prominent part in the work of the local friendly societies, and for 14 years has been a member of the Guildford Institute, in addition to holding many other honorary official posts in the borough.

It is, however, more especially in connection with the work of the Royal Surrey County Hospital that Mr. Patrick's name has been prominently before the public. Appointed in 1900 to the post of Assistant Secretary (the Honorary Secretaryship is held by Mr. Edward Gibson, J.P.), Mr. Patrick has given zealously of his time, his interest, and his abilities to the institution. During his association with the hospital the finances have largely improved, and many additions to the structure and equipment have been effected. A new $x$-ray department has been installed by a prominent supporter of the institution, who gave £1,000 for this and other additions, new kitchens and dining-rooms have been erected, the accommodation of the Nurses' Home has been considerably increased, a ward closed through want of funds has been added to the bed accommodation, and a "pound day" has been successfully established. But the greatest asset to the institution is the building and equipping of a new Convalescent Home at Worthing, provided for by the late Mr. George Prentice Shepherd, a generous and timely benefactor of the hospital. The total cost of the Home was £3,100, £3,000 of which was provided out of Mr. Shepherd's endowment, the balance of £7,000 being added to the Endowment Fund of the hospital.

In acknowledging the honour conferred upon him by his colleagues on the Borough Council, Councillor Patrick remarked: "It had been his happy privilege to be associated for some 10 years with one of the largest institutions in the borough—the Royal Surrey County Hospital. He might call the institution a happy hunting-ground for mayors. It was surprising the number of gentlemen who had filled the office of mayor who had been connected with the hospital on the Board of Management, and at the present time he believed there were several past mayors on the board. He would like to be allowed to mention the arrangements that he had been permitted to make with the Board of Management of the hospital for the fulfilment of his Secretarial duties during his mayoralty. He was to appoint a *locum tenens* during the year of his office, so that he might be free to carry out as far as possible the arduous duties which now devolved upon the Mayoralty.

Councillor Patrick's duties will be very heavy during the Coronation year, and he has already outlined a tea for the old people and the children, which he thinks will meet with general approval.

Hospital officials generally will wish Councillor Patrick unqualified success in all his efforts, health and strength to carry out his important duties, and that at the end of the mayoral term Councillor and Mrs. Patrick will have the happiest reflections on the work they have been able to accomplish.

### DEATH OF A BENEFACTOR OF GUY'S.

There has lately passed away a very gracious lady and generous benefactress of Guy's Hospital, Miss Louisa Ann Williams. We are indebted to the *Hospital Gazette* for the following interesting sketch of this lady's career as a hospital philanthropist :—

"Miss Williams first became known to Guy's through her letter of March 17, 1897, inquiring particulars as to the endowment of beds, and intimating her desire to perpetuate in some way the memory of her friend, Henry Pratt, M.D., sometime a student of Guy's Hospital. The reply to that letter was quickly followed by a cheque for £1,000, and the Henry Pratt Bed is now No. 29 in Astley Cooper Ward. Miss Williams was a stranger to the hospital; at no time was she within its gates; but the interest in the institution, originally aroused in her mind by her old friend, stimulated by the urgent appeal then being made on its behalf, thenceforth never waned. Although an elderly lady, she maintained with the Secretary to the Treasurer a correspondence marked by the clear vision of an able and generous mind; and some three months after her first donation she spontaneously conveyed to the Re-endowment Fund freehold property at Leamington to the value of £3,000. This was followed at later dates by donations amounting in all to £5,000 for the endowment of five beds. These beds, bearing the simple identification 'L. A. W.,' are No. 10, Barnabas; No. 4, Cornelius; No. 4, John; No. 14, Luke; and No. 1, Lydia.

"Friendly communications continued to be received from Miss Williams during subsequent years, and at her request she was kept informed of the hospital's condition. Then, in 1906, came the climax of her beneficence, when the Re-endowment Fund received from her property and investments to the value of £16,682.

"As the final result of Miss Williams' dispositions the hospital has benefited from first to last to the extent of some £29,000, an offering entirely voluntary and unsolicited.

"Miss Williams was a remarkable woman. Cultured, travelled, possessed of high powers of understanding and knowledge of the world, but above all simple and unselfish. Born at Malden Rectory, Surrey, in 1821, the daughter of the Rev. Henry Williams, she survived all her relations and friends of early life, and, having lived in many places, came at last to Ross, in Herefordshire, and there, near by the hospital's estate, she spent her closing years, with a mind cheerful, serene, and unclouded almost to her death, which took place on the 10th ultimo in the ninetieth year of her age. Her last letter, written on September 22 to the Clerk to the Governors, says : 'I send you many many thanks for the two lovely boxes of roses I have received. I am not any better, but not in a suffering condition. I hope all good things to the hospital are prospering. This is all I can do.'

"Certainly, Miss Williams had done what she could and never asked even the smallest privilege in return beyond on one occasion—she was a Roman Catholic—gently expressing the desire that a crucifix might be placed somewhere in the hospital 'among the sick and suffering' for their 'consolation, comfort, and support'; and none will take offence at the beautiful crucifix from Oberammergau which hangs in the Mortuary Chapel. Miss Williams was laid to rest in the lovely churchyard of Ross on October 19 in the presence of Mr. Henry Williams and Mr. Charles H. Wells on behalf of the hospital, and of those who had cared for her in her last illness."

### Personalia.

Every supporter of the Trowbridge Cottage Hospital will realise the loss suffered by the institution through the death of Mrs. Gouldsmith; and it is satisfactory to note that a record of its debt to her has been embodied in the annual report. Her late husband, Mr. Jesse Gouldsmith, was the founder of the hospital, and his family has been in the most intimate connection with the hospital ever since. Mrs. Gouldsmith particularly spared no generosity or pains on its behalf, and a hospital, especially at a moment when it has organised successfully a new scheme of management, cannot do better than foster the family traditions of the past.

Mr. E. Hastings Tweedy's seventh and final year of office as Master of the Rotunda Hospital, Dublin, having expired, the following resolution was passed unanimously : "That the Board of Governors of the Rotunda Hospital, on the retirement of Dr. Ernest Hastings Tweedy from the office of Master of the hospital, sincerely tender to him our warm thanks for his devoted attention to the patients and to the interests of the charity, and we desire to assure him of our appreciation of the distinguished skill which he has shown during his term of office, and most warmly congratulate him on the great efficiency of the hospital, and on the improvements in the institution which have been effected during his mastership, in the carrying out of which he has so materially assisted. We further desire to convey to him our best wishes for his success in his future career."

The Agnew family has been connected with the Manchester Children's Hospital, Pendlebury, since the early seventies, and the late Sir William Agnew, Bart., was for a long time President of that institution. At the meeting of the Board of Governors held last week the following resolution was unanimously passed : "The Governors of the Manchester Children's Hospital have received with deep regret the news of the death of Sir William Agnew, Bart., their President since 1892. Sir William Agnew's connection with the Hospital extended over a period of more than thirty years, during which time his interest in its affairs has been unfailing and his generosity continuous. Among his many gifts the Governors especially recall with gratitude that of the Convalescent Home at St. Anne's, which has been the means of restoring to health hundreds of children who, but for it, would have had no chance of making a satisfactory recovery. The Governors are well aware that by his death many good causes will lose a valued friend, but they do not think that in any connection have his services been more highly appreciated, nor will his loss be more severely felt, than by those responsible for the maintenance of the hospital. They desire to offer to his family an expression of their sincere sympathy."

### Elections and Appointments.

Mr. William Plunket Cairnes, of Stameen, has been elected a vice-president of the Board of Governors of the Rotunda Hospital, Dublin. Mr. Richard S. Reeves has been elected a governor; Sir Algernon Coote, Bart., H.M.L., and Mr. E. Hastings Tweedy have been proposed for election as governors at the next meeting.

